# Rapid Progression of Large B-cell Lymphoma in Behçet's Disease on Immunosuppressive Therapy: A Case Report with Literature Review

**DOI:** 10.7759/cureus.28029

**Published:** 2022-08-15

**Authors:** Ashley Aya, Amanda Dawson, Palak Patel, Cristina L Acosta, Anna Dedona

**Affiliations:** 1 Internal Medicine, Hackensack Meridian Ocean Medical Center, Brick, USA; 2 Medicine, Saint George's University, Grenada, USA

**Keywords:** non-hodgkins lymphoma, t-cell/histiocyte-rich, lymphoproliferative disorder, cancer, immunosuppressive therapy, vasculitides, b-cell lymphoma, systemic vasculitis, behçet's disease

## Abstract

Behçet's disease (BD) is a systemic vasculitis characterized by various symptoms, including orogenital ulcers, uveitis, arthritis, skin lesions, and the involvement of the gastrointestinal tract and central nervous system. BD has been associated with malignancies such as leukemia, myelodysplastic syndrome, lymphoma, multiple myeloma, Hodgkin's disease, and lymphosarcoma. The rarity of association with B-cell lymphoma may also be added to the list, given our findings in this case report. Patients with vasculitides benefit from immunosuppressive therapy that can minimize disease and may prevent disease manifestations and exacerbations. However, there may be an increased risk of cancer development, which calls for consideration while starting and maintaining this population of patients on immunosuppressive therapy.

## Introduction

Behçet's disease (BD) is a chronic, systemic immune-mediated vasculitis affecting the vascular, gastrointestinal, neurological, eye, and joint involvement. It is characterized by painful aphthous ulcers, genital mucosal ulcers, uveitis, and dermal lesions. The clinical triad of hypopyon, iritis, and orogenital ulcers was described by the Turkish dermatologist Hulusi Behçet in 1937 as a chronic and consistent presentation among three of his patients. After gathering the details and commonalities of the symptoms from those patients and a non-diagnostic biopsy from each, he recognized the cluster of symptoms as a possible manifestation of a new disease with a viral basis, thus assigning the name "Behçet's disease." The disease was possibly initially described back in 470-370 BC by the Greek physician Hippocrates of Kos, who discovered the presentation of aphthous ulcers during an endemic in Asia Minor at that time [1]. One of the most prominent associations among BD patients is a geographic location along the Silk Road countries, a collection of ancient trading routes extending from southern Europe to East Asia. Turkey has the most significant BD prevalence, ranging from 80 to 420 per 100,000. According to studies, the prevalence of BD is also high in East Asia, with 13.5 per 100,000 in Japan and 14.0 per 100,000 in China. Northern Europe, western Europe, and the United States have recorded low prevalence [2]. The importance of genetic variables in the etiology of BD is supported by familial aggregation of the disease, which differs among populations. The familial collection is more extensive among Turks (18.2%), Koreans (15.4%), and Jews (13.2%) than among Chinese (2.6%), Japanese (2.2%), and different European ethnicities (0-4.5%) [2]. Currently, the disease is known to have an incidence of one to two people per 1,000,000 in the North American and UK populations. The age of incidence ranges from 20 to 40 years [1]. The standard treatment of BD is corticosteroids (CS) and immunosuppressants such as tumor necrosis factor (TNF) inhibitors (TNF-i). The cure rate of predominant symptoms of BD at one year is 60.0% (12 of 20 patients) with TNF-i and CS therapy [3].

Here we describe the case of a 54-year-old female with a long-standing history of BD, treated empirically with immunosuppressants and then presented with three weeks of dyspnea to our ED, found to have multiple PE and an adrenal mass biopsy that revealed a T-cell/histiocyte-rich large B-cell lymphoma.

## Case presentation

A 54-year-old female presented to the hospital with the complaint of shortness of breath for the previous three weeks. Her dyspnea was associated with fever, chills, rigors, nonproductive cough, and chest pain. On the day of the presentation, the patient was seen by her primary care physician (PCP) for a sick visit. She had a chest X-ray done in the office that showed pulmonary nodules. Her PCP subsequently sent her to the emergency department (ED) to evaluate her worsening dyspnea with concern for pulmonary embolism. She was a nonsmoker. She has a past medical history of BD, cerebral vascular accident, hypertension, hyperlipidemia, asthma, chronic pancreatitis, and gastroparesis. She had a longstanding history of BD complicated with oral ulcers, joint pain, and stroke and had been treated with azathioprine for over four years. 

**Figure 1:**
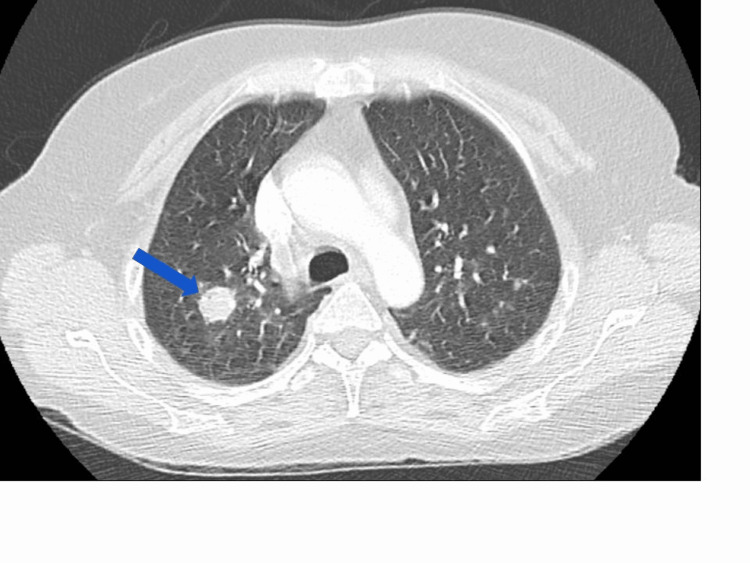
CT angiography of the chest showing the largest right lower lobe nodule (blue arrow).

Her vitals in the ED were as follows: blood pressure 99/57 mmHg, pulse 109 beats per minute, respiratory rate 24 breaths per minute, saturating 100% on room air, and temperature 101.6 degrees Fahrenheit. The physical exam was non-contributory. Laboratory revealed white blood cells (WBC) count 5.4 k/uL (4.5-11), hemoglobin 10.5 g/dL (12-16), creatinine 1.89 mg/dL (0.44-1.00), and sodium 131 mmol/L (136-145). Imaging studies -- CT angiogram of the chest showed multiple filling defects in the subsegmental arteries of the right middle lobe, right lower lobe, left lower lobe, and multiple pulmonary nodules scattered throughout the lungs, with the largest in the right lower lobe measuring 36 mm in size (Figure 1), a 38.5 mm x 28.6 mm left adrenal nodule, and right paratracheal adenopathy with the largest lymph node measuring 18.9 mm. The lung and left adrenal nodules were not present on CT imaging about five months before presentation. Doppler ultrasound of the lower extremities was negative for deep vein thrombosis.

Given these findings, the patient was treated with heparin for bilateral pulmonary emboli and admitted to the intensive care unit (ICU). The multiple lung nodules and left adrenal nodules were suspicious of malignancy. However, further testing was put on hold in lieu of acute treatment of her pulmonary emboli. Over the next week, the patient decompensated and went into acute hypoxic respiratory failure with eventual intubation. This was followed by acute respiratory distress syndrome requiring a RotoProne bed, acute kidney injury leading to dialysis, and acute chronic pancytopenia. Additional laboratory results revealed a CD4 count of 39 cells/uL. However, HIV 1 and HIV 2 were not detected. She had an extensive autoimmune workup that was negative. Nonetheless, she was started on steroids for concern of acute vasculitis flare. Adrenal gland biopsy was conducted during the hospital admission, and pathology revealed T-cell/histiocyte-rich large B-cell lymphoma. This lymphoma was found to be compatible with the immunodeficiency-associated lymphoproliferative disorder (LPD).

## Discussion

Autoimmune and autoinflammatory diseases are present in BD [2]. Clinical factors and organ systems in each patient's situation direct the treatment of choice. According to studies, BD increases the risk of cancer, specifically non-lymphoma, Hodgkin's lymphoma, myeloma, and leukemia [4]. While BD may not always cause cancer, there is evidence that BD treatment can be.

At this moment, the cause of Behçet's illness is unknown. Differences in environmental and genetic factors are thought to cause the large variety of illness prevalence reported across different geographic regions. New genetic markers and lymphocyte function, notably T-helper cells, have been highlighted in recent research. BD is frequently connected with the human leukocyte antigen-B51 (HLA-B51) allele of the major histocompatibility complex (MHC). According to several studies, 40%- 80% of patients with BD have HLA-B51, whereas only 10%-30% of controls have HLA-B51 [1-2, 4]. This indicates that these genes may be directly responsible for BD and serve as persuasive diagnostic criteria.

Another hypothesis arises in the link between BD and infectious agents such as *Streptococcus sanguis*. The *S. sanguis* antigens in the skin and sanguineous monocytes will elicit a hypersensitivity response in patients with BD. Other contagious agents associated with BD include the herpes simplex virus (HSV), given the antibodies to HSV-1 and circulating immune complexes found in patients with BD [1]. 

The most commonly associated malignancies include non-Hodgkin’s lymphoma (NHL), Hodgkin’s lymphoma, and leukemia (Table 1). NHL has an incidence 60% higher among patients with autoimmune diseases than in the general population. Studies reveal that NHL has a positive association with 21 specific autoimmune diseases; BD is included with a standardized incidence ratio (SIR) of 1.7. Hodgkin's Lymphoma is positively associated with 11 autoimmune diseases, including BD (SIR=5.6) [4].

**Table 1:** Most commonly associated malignancies.

Malignancy
Non-Hodgkin's lymphoma
Hodgkin's lymphoma
Leukemia
Oral cavity and pharyngeal cancer
Thyroid cancer

Population research conducted in Korea, a country with a high prevalence of BD, found that the crude cancer incidence rates per 1000 people in the BD and control groups were 5.54 and 4.86, respectively. With a hazard ratio (HR) of 5.801, patients with BD were at higher risk for leukemia than controls (95% CI, 3.24-10.385). Lymphoma was more common in patients with BD (HR= 1.784, 95% CI, 1.141-2.791) [5].

Aside from their role in BD treatment, various studies have linked immunosuppressants to a higher likelihood of malignancy. Dantal et al. reported one of the most straightforward pieces of evidence for this link in a randomized study of two cyclosporine regimens in renal transplant patients (low dose with blood concentrations of 75-125 ng/mL) and normal dose with blood concentrations of 150-250 ng/mL). After 66 months of follow-up, the normal dose group of patients was found to have a higher rate of cancer diagnoses. Other research has found that combining cyclosporine with azathioprine and CS increases the risk of cancer, especially non-melanoma skin tumors [6-8].

B-cell lymphoma has not been recognized as a common finding of the malignancies reported in association with BD. There is minimal research tailored to the link between the two. This case report is one of the first to arise in evidence of just how rare B-cell lymphoma is in patients with BD. 

According to the World Health Organization, T-cell/histiocyte-rich sizeable B-cell lymphoma is characterized by less than 10% of large neoplastic B-cells within a background of abundant T-cells and frequent histiocytes. Identifying this variant is essential as the cases containing numerous histiocytes behave aggressively and show resistance to current therapies for diffuse large B-cell lymphoma [9]. They can be identified based on morphological and immunohistochemical analysis of neoplastic cells and non-neoplastic stromal or nonstromal background [9]. In these immunodeficiency-associated LPDs, the traditional distinction between polymorphic and monomorphic LPDs is obscure and non-reproducible [10]. In immunodeficiency-associated large B-cell lymphomas with a mixed background of increased T-cells and histiocytes, classification as Hodgkin lymphoma vs. non-Hodgkin lymphoma is often tricky and ambiguous. These lymphomas represent a histopathologic and immunophenotypic continuum between T-cell/histiocyte-rich B-cell lymphoma and classical Hodgkin’s lymphoma [11]. The T-cell/histiocyte-rich subtype of large B-cell lymphoma is associated with diagnosing BD and azathioprine therapy. In this case, the report is not a usual finding among patients with BD.

Behçet's disease typically presents in patients between 20 and 40 years old. The course of BD is chronic and generally includes bouts of remissions and exacerbations. All cases will present with painful oral ulcers (2-10 mm in size); other symptoms depend on the systems affected (Table 2). Genital ulcers will be similar in appearance to oral ulcers. Some patients may exhibit cutaneous manifestations such as nodular lesions and erythema nodosum-like lesions. Ocular involvement may be evident in decreased or blurry vision. Patients may describe arthralgias. Patients may also present with hypertension and dyslipidemia.

**Table 2:** Physical exam findings and clinical features of BD. BD, Behçet's disease

System	Physical findings and clinical features
Oral/Gastrointestinal	Painful aphthous ulcers
Genitourinary	Recurrent genital mucosal ulcers similar to oral ulcers
Rheumatologic	Arthritis, vasculitis
Dermatologic	Nodular lesions, erythema nodosum-like lesions, papulopustular lesions, positive pathergy test
Ocular	Anterior/posterior veitis, hypopyon, keratitis, iritis, vitreous hemorrhage or occlusion
Vascular	Superficial and deep-vein thrombosis, Budd-Chiari syndrome, inferior vena cava syndrome, pulmonary artery involvement, and cerebral venous sinus thrombosis
Neurologic	Brainstem involvement, myelitis, pseudotumors

The pathergy test, genetic markers, and histopathology in conjunction with clinical criteria are used in diagnosing BD, as the condition lacks pathognomonic symptoms and laboratory findings. The International Study Group for Behçet's Disease has developed the most widely accepted clinical criteria for diagnosing BD. Recurrent oral ulcers with two bars are required: recurrent vaginal ulcers, eye lesions, skin lesions, or a positive pathergy test. The pathergy test uses a sterile 20-22 gage needle to penetrate the skin to a depth of 5 mm obliquely. A positive result is if an erythematous papule emerges at the test site after 48 h [1]. Nodular cutaneous lesions, papulopustular lesions, and mucocutaneous aphthae are seen on gross examination. Microscopically, significant neutrophil infiltration is seen in all early lesions. Lymphocytic infiltrates and liquefaction-degeneration is seen at the dermal-epidermal junction in mucocutaneous lesions [1]. Vascular endothelium reveals endothelial cell swelling and fibrinoid necrosis. As previously mentioned, studies show a high frequency of HLA-B51 allele carriage in the MHC region on chromosome 6p21 in most, but not all, patients with BD. This allele is likely found in patients with more severe disease complications [2]. 

Serum results may show elevated cytokines such as TNF, IL-1b, and IL-8, indicating the pro-inflammatory state consistent with BD. Inflammatory markers that may also be significantly elevated include C-reactive protein (CRP) and C3. Findings of anti-endothelial cell antibodies may be elevated, indicating the presence of vasculitides [1-2].

Treatment for BD is symptomatic and based on the system involved. The main goal of treatment is to prevent relapses and reduce inflammation in vital organs that could result in irreversible damage. The mainstay of therapy is CS combined with other immunosuppressive agents for induction or maintenance of treatment. Other drugs used are colchicine, azathioprine (AZA), cyclosporine-A (CSA), cyclophosphamide, interferon (IFN) alpha, and TNF-i [12].

Appropriate treatment of mucocutaneous lesions includes topical CS, colchicine, thalidomide, dapsone, and interleukin-1 inhibitors (IL-1i) such as canakinumab and anakinra. Colchicine is proven most effective for genital ulcers, specifically in female patients. Thalidomide may cause erythema nodosum lesions to flare up. AZA, etanercept (TNF-i), and IFN alpha [12] can also be used to treat mucocutaneous lesions. 

Acute onset of neuro-Behçet’s disease (NBD) is treated with CS and IV cyclophosphamide or azathioprine. Chronic NBD, which is more severe than the sensitive type, receives the most significant benefit from TNF-i (infliximab) [6, 12]. CS should be used as well as sulfasalazine or AZA for acute exacerbation of symptoms. Infliximab and thalidomide are acceptable treatments for refractory patients [12]. 

If left untreated, ocular involvement might result in irreversible visual loss. Isolated anterior uveitis can be treated with topical agents, and high-dose glucocorticoids are used in patients with acute posterior uveitis. AZA is recommended in patients with hypopyon. Cyclosporine-A effectively decreases the frequency and severity of ocular attacks [12].

Corticosteroids and AZA or CsA are recommended in patients with acute deep vein thrombosis. Anticoagulants are considered for refractory cases and patients with more extensive vein thrombosis or Budd-Chiari syndrome. High-dose glucocorticoid and cyclophosphamide are recommended for patients with pulmonary artery aneurysms. AZA should be given as maintenance therapy [12].

Colchicine is proven effective in the initial treatment. Intra-articular glucocorticoids can be used if the symptoms are monoarticular. An acute exacerbation of symptoms may be alleviated by non-steroidal anti-inflammatory drugs [12]. The presence of anterior uveitis, young age, early-onset, and male gender have all been found to be poor predictive factors in studies. Other clinical variables can indicate poor prognoses, such as the absence of mucosal healing in patients with ulcers, elevated CRP, and lack of initial response to therapy. Neurological involvement is also associated with poor prognosis. NBD patients with the HLA-B51 allele experience a worse prognosis [6, 8, 12]. NBD patients receiving IV cyclophosphamide have a more prolonged event-free survival.

## Conclusions

Behçet’s disease is a chronic, systemic immune-mediated vasculitis. The autoimmune component of BD is significant in its correlation with malignancies. Previous studies have shown an elevated risk of hematologic malignancies, in particular. The mainstay of therapy for patients with BD is CS in combination with other immunosuppressive agents for induction or maintenance of therapy, such as azathioprine or cyclosporine. The use of immunosuppressants in the treatment of BD further increases a patient's susceptibility to cancer, and in this case, immunodeficiency-associated large B-cell lymphoma.
